# Supplementary material for: Haploid mouse germ cell precursors from embryonic stem cells reveal Xist activation from a single X chromosome
Source: Stem Cell Reports. 2021 Dec 16;17(1):43–52. doi: 10.1016/j.stemcr.2021.11.006 (PMC8758942; doi:10.1016/j.stemcr.2021.11.006)
Supplement: Document S1. Supplemental experimental procedures, Figures S1 and S2, and Table S1 [file mmc1.pdf]

Stem Cell Reports, Volume *17*

Supplemental Information

Haploid mouse germ cell precursors from embryonic stem cells reveal *Xist* activation from a single X chromosome

Eishi Aizawa, Corinne Kaufmann, Sarah Sting, Sarah Boigner, Remo Freimann, Giulio Di Minin, and Anton Wutz

Figure S1

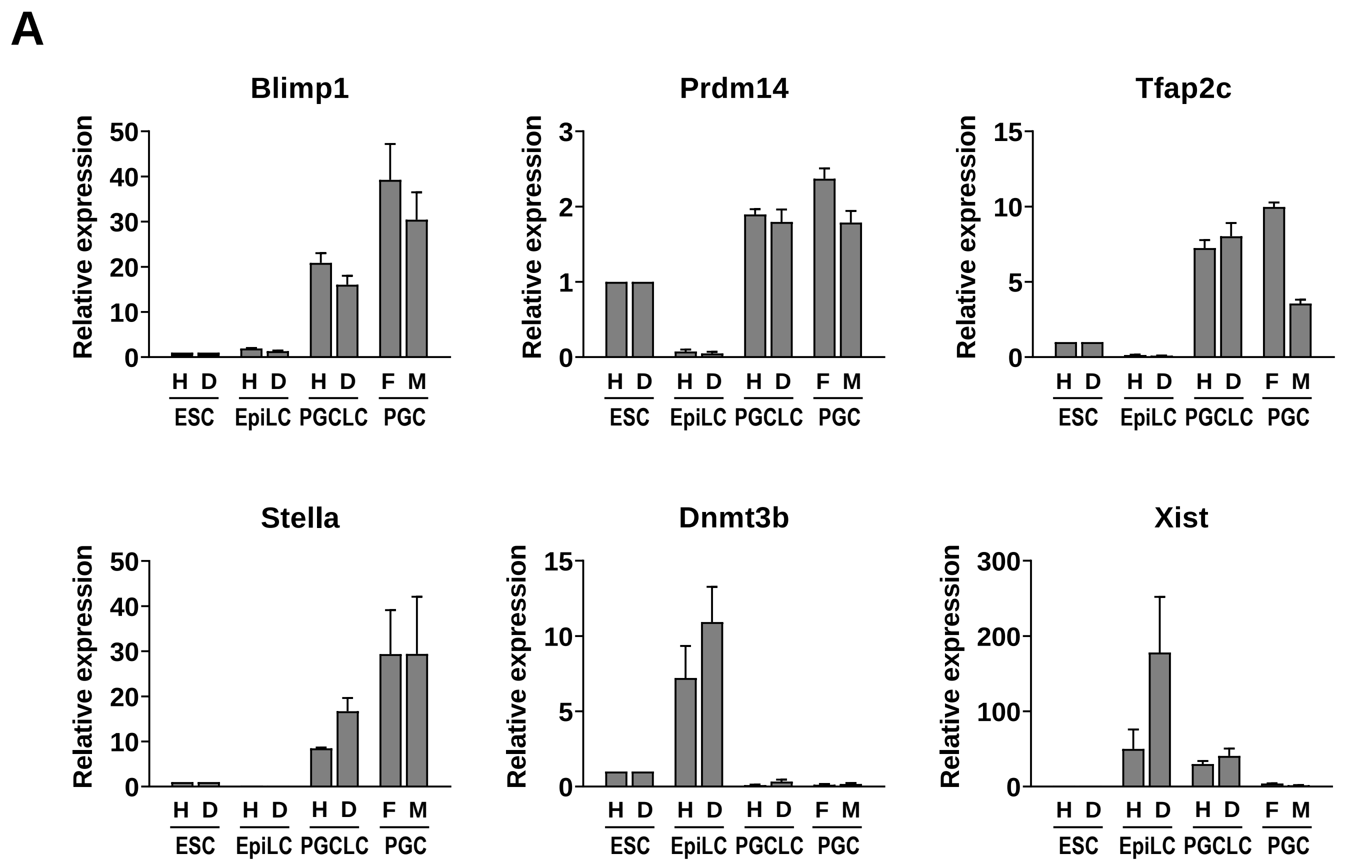
A
Blimp1
Prdm14
Tfap2c
Stella
Dnmt3b
Xist
Relative expression
H D
F M
ESC
EpiLC
PGCLC
PGC

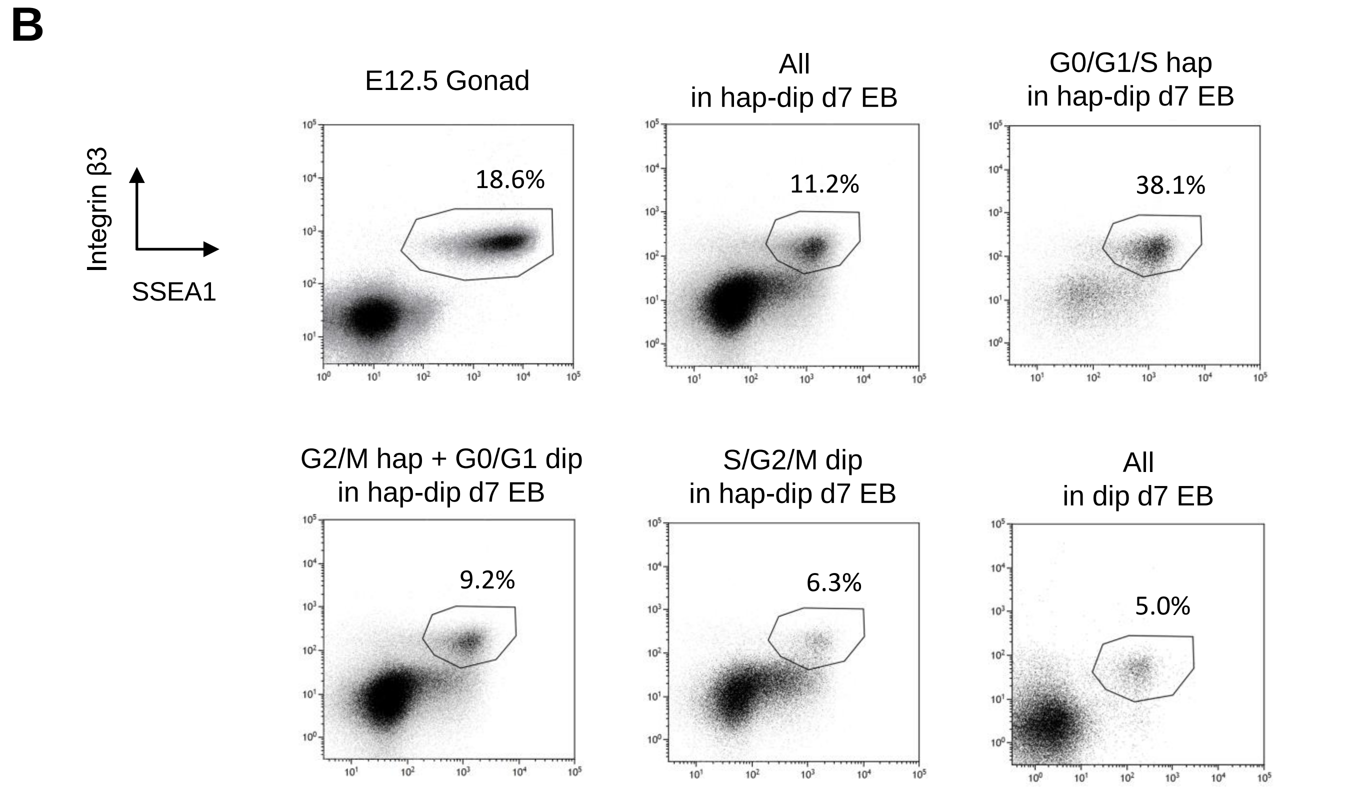
B
Integrin β3
SSEA1
E12.5 Gonad
18.6%
All
in hap-dip d7 EB
11.2%
G0/G1/S hap
in hap-dip d7 EB
38.1%
G2/M hap + G0/G1 dip
in hap-dip d7 EB
9.2%
S/G2/M dip
in hap-dip d7 EB
6.3%
All
in dip d7 EB
5.0%

Figure S2

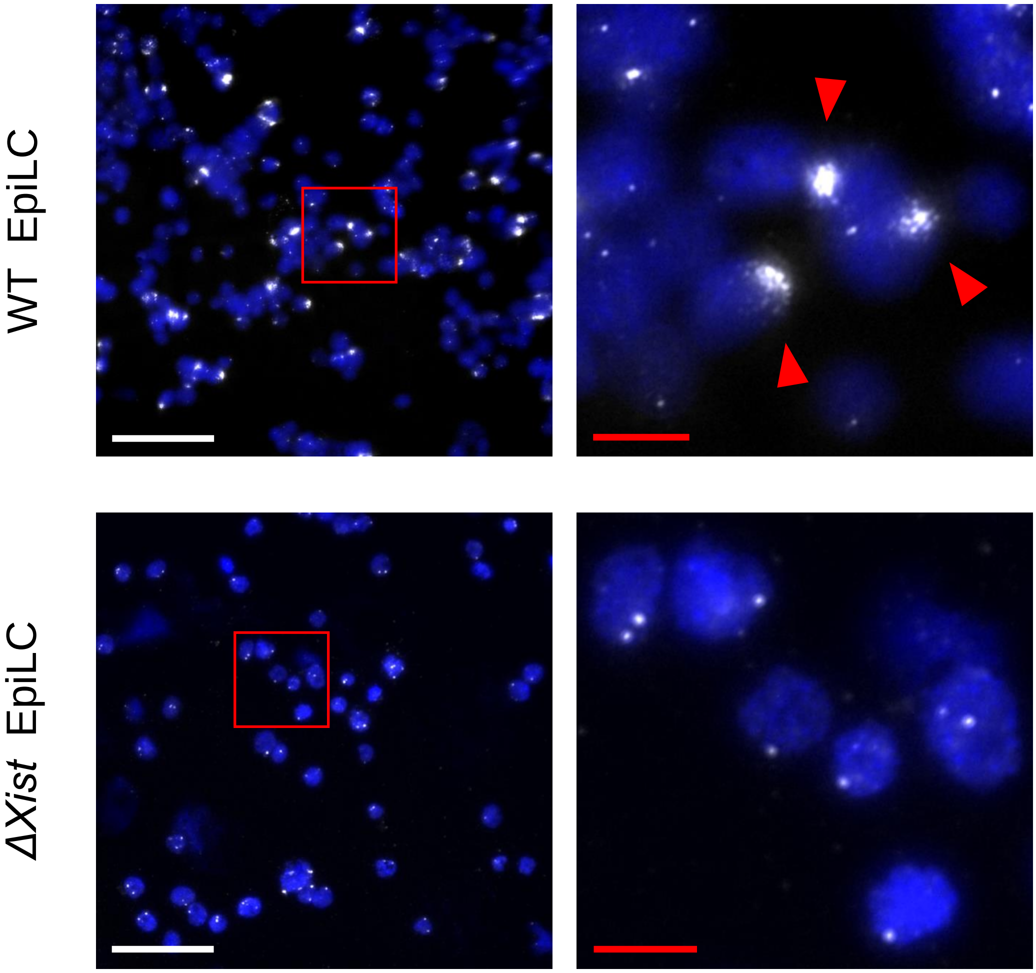

Supplemental Figure Legends

Figure S1. Transcription profile and flow cytometry analysis during germ cell differentiation *in vitro*, related to Figures 1-3. (A) Transcription of PGC markers (*Blimp1*, *Prdm14*, *Tfap2c* and *Stella*), *Dnmt3b* and *Xist* during germ cell differentiation of haploid-diploid mixed ESCs. Gene expression of G0/G1/S-phase haploid and S/G2/M-phase diploid cells of ESCs, EpiLCs and d7 EBs was measured, respectively. Female and male PGCs purified from E12.5 gonads by FACS were used as controls. Gene expression of G0/G1/S-phase haploid and S/G2/M-phase diploid samples was normalized to *Gapdh* expression relative to G0/G1/S-phase haploid and S/G2/M-phase diploid ESCs, respectively. Gene expression of male and female PGCs was normalized to *Gapdh* expression relative to S/G2/M-phase diploid ESCs. Data are derived from 4 biological replicates of each sample. The data represents relative expression with the mean value and the standard error of the mean. H, G0/G1/S-phase haploid; D, S/G2/M-phase diploid; F, female; M, male. (B) A representative flow cytometry analysis of d7 EBs. PGCLCs accounted for 11.2% out of all cells in d7 EBs derived from haploid-diploid mixed ESCs. PGCLCs accounted for 38.1%, 9.2% and 6.3% out of G0/G1/S haploid cell population, G2/M haploid + G0/G1 diploid cell population, and S/G2/M diploid cell population of d7 EBs derived from haploid-diploid mixed ESCs, respectively. Embryonic female gonads at E12.5 and d7 EBs derived from diploid ESCs were also analyzed as controls.

Figure S2. Effect of *Xist* deletion on RNA FISH using a *Xist* probe, related to Figure 4. Representative images of WT and *ΔXist* haploid-diploid mixed ESCs after EpiLC

differentiation for 48 hours. *Xist* expression was detected by RNA FISH using a *Xist* probe (white). Nuclei are shown in blue (DAPI staining). The area marked by a red square in the left image is enlarged on the right. *Xist* clusters were observed in WT EpiLCs (arrowheads) but not in *ΔXist* EpiLCs.Scale bar (white), 100 μm; scale bar (red), 20 μm.

Table S1. List of oligos

Name	Sequence (5' to 3')	Application
Blimp1-F	AGC ATG ACC TGA CAT TGA CAC C	RT-PCR
Blimp1-R	CTC AAC ACT CTC ATG TAA GAG GC	RT-PCR
Dnmt3b-F	CTC GCA AGG TGT GGG CTT TTG TAAC	RT-PCR
Dnmt3b-R	CTG GGC ATC TGT CAT CTT TGC ACC	RT-PCR
Gapdh-1	AGG TCG GTG TGA ACG GAT TTG	RT-PCR
Gapdh-2	TGT AGA CCA TGT AGT TGA GGT CA	RT-PCR
Prdm14-F	ACA GCC AAG CAA TTT GCA CTA C	RT-PCR
Prdm14-R	TTA CCT GGC ATT TTC ATT GCT C	RT-PCR
Stella-F	AGG CTC GAA GGA AAT GAG TTT G	RT-PCR
Stella-R	TCC TAA TTC TTC CCG ATT TTC G	RT-PCR
Tfap2c-F	GGG CTT TTC TCT CTT GGC TGG T	RT-PCR
Tfap2c-R	TCC ACA CGT CAC CCA CAC AA	RT-PCR
Xist-F	GCT GGT TCG TCT ATC TTG TGG GTC	RT-PCR
Xist-R	TTG TTC AGA GTA GCG AGG ACT TGA AGA G	RT-PCR
Xist-P1	CCA GCC ATG TTT GCT CGT TT	Genotyping
Xist-P2	GGC GAA GGA GTA TGG CCT TT	Genotyping
Xist-gRNA1-F	CACC AAG CCA TAA GGC TTG GTG GT	gRNA
Xist-gRNA1-R	AAAC ACC ACC AAG CCT TAT GGC TT	gRNA
Xist-gRNA2-F	CACC GAC CCT TGC TGT ACT GCA AA	gRNA
Xist-gRNA2-R	AAAC TTT GCA GTA CAG CAA GGG TC	gRNA

Supplemental Experimental Procedures

Derivation of a *ΔXist* haESC line

For derivation of a *ΔXist* haESC line, a deletion of *Xist* exon 1 was engineered in a haESC line from 129S6/SvEvTac mice using the CRISPR-Cas9 system. Two guide RNAs (gRNAs) targeting *Xist* exon 1 were designed using Synthego CRISPR Design Tool (www.synthego.com). Sequences of gRNAs are listed in Table S1. The gRNAs were ligated into the pX330-U6-Chimeric_BB-CBh-hSpCas9 vector (Addgene, #42230) that was digested with BbsI restriction enzyme. Transfection of vectors and the establishment of targeted haESC lines were performed as previously described (Aizawa et al., 2020a; Aizawa et al., 2020b). Briefly, 2 Cas9/gRNA vectors, a piggyBac plasmid carrying a CAG-DsRed-IRES-hygro transgene, and a hyperactive piggyBac transposase plasmid were transfected into the haESC line using lipofectamine 2000 by following the manufacturer's protocol. Subsequently, single haploid cells expressing DsRed were isolated by cell sorting (MoFlo Astrios EQ, Beckman Coulter) after staining with 15 µg/ml Hoechst 33342 (Invitrogen) and cultured on irradiated MEFs in Serum+2i+LIF medium. After the growth of clonal single colonies, a subset of cells in each line was analyzed by flow cytometry after staining with Hoechst 33342 to select cell lines containing haploid cells and genotyped to screen cell lines for a deletion of *Xist* exon 1. Subsequently, the haploid 1n cell population of each selected haploid cell line was purified by cell sorting after Hoechst 33342 staining and was cultured on a gelatin-coated plate without MEFs in Serum+2i+LIF medium.

In vitro neural lineage differentiation

The differentiation procedure to derive neural stem cell-like cells (NSCLCs) from haESCs

was performed following a published protocol (He et al., 2017) with a few modifications as schematically summarized in Figure 4E. Briefly, at day 0 haploid-diploid mixed ESCs were plated into a Sphericalplate 5D (Kugelmeiers Ltd.) in N2B27 medium supplemented with 300 nM LDN193189 (MedChemExpress, HY-12071A), 10 μM SB431542 (Stemgent, 04-0010-10) and 20 μM Y-27632 (ROCKi; Tocris, 1254). After 3 days, the EBs were plated onto dishes coated with 50 μg/ml poly-D-lysine and 10 μg/ml laminin in NSCLC differentiation medium, which consists of N2B27 medium supplemented with 300 nM LDN193189, 10 μM SB431542, 20 μM Y-27632, 20 ng/ml EGF (Peprotech, 315-09), and 20 ng/ml FGF2 (Peprotech, 450-33). On day 5, cells were washed and NSCLC differentiation medium without Y-27632 was added. At day 7 after initial induction, the differentiated cells were dissociated, and their DNA content was measured by flow cytometry.

Karyotyping

On day 7 of germ cell differentiation, M-phase arrest of EBs was performed by culturing in PGCLC differentiation medium supplemented with 0.05 mg/ml demecolcine (Merck) for 8 hours. Subsequently, PGCLCs were sorted by FACS. Chromosome counting of PGCLCs was performed as previously described (Aizawa *et al.*, 2020a).

Genotyping

Genotyping of ESC lines was performed as previously described (Aizawa *et al.*, 2020a; Aizawa *et al.*, 2020b). Sequences of *Xist* exon 1 were obtained from PCR products through the commercial Ecoli NightSeq service (Microsynth). Primers used for genotyping are listed in Table S1.

Transcription analysis

During germ cell differentiation of haploid-diploid mixed ESCs, G0/G1/S-phase haploid and S/G2/M-phase diploid ESCs, EpiLCs, and d7 PGCLCs were sorted from ESCs, EpiLCs and d7 EBs by FACS, respectively. E12.5 gonads were harvested from 129S6/SvEvTac mouse embryos. Sex of the gonads was determined by their morphology. PGCs were identified as gonadal cells positive for both SSEA1 and integrin β3 and were sorted by FACS. RNA of each sample was extracted using the RNeasy Mini Kit (Qiagen) following the manufacturer's protocol, including an on-column DNA digest using RNase-free DNase (Qiagen). RNA concentration was determined using a NanoDrop Lite (Thermo Fisher Scientific). 100 - 500 ng total RNA was reverse transcribed using the PrimeScript RT Master Mix (Takara) according to the manufacturer's instruction. RT-PCR was performed on a 384 well format with the 480 Lightcycler instrument (Roche) using KAPA SYBR FAST qPCR KIT (Kapa Biosystems). Fold change expression was calculated using the ΔΔct method. *Gapdh* expression of G0/G1/S-phase haploid ESCs was used to normalize the transcription of G0/G1/S-phase EpiLCs and d7 PGCLCs. *Gapdh* expression of S/G2/M-phase diploid ESCs was used to normalize the transcription of S/G2/M-phase EpiLCs, d7 PGCLCs and PGCs. Primers used for transcription analysis are listed in Table S1.

Xist RNA FISH

RNA FISH was performed to analyze *Xist* expression during differentiation of haploid-diploid mixed ESCs. The *Xist* FISH probe was prepared from the ptetOP-Xist-PA plasmid with Cy3-dCTP (Amersham Biosciences) as described previously (Wutz and Jaenisch, 2000). For analysis of germ cell differentiation, G0/G1/S-phase haploid and S/G2/M-phase diploid ESCs, EpiLCs and d7 PGCLCs were sorted by FACS, respectively. For analysis of

time frame EpiLC and *Xist* deletion effect, all cells containing both haploid and diploid population were used after harvesting without cell sorting. Cells were mounted onto glass slides using a Cytospin 4 (Thermo Scientific) for 3 min at 800 or 900 rpm for haploid or diploid cells, respectively. Glass slides were immediately rinsed in PBS and washed in CSK buffer (100 mM NaCl, 300 mM sucrose, 3 mM $MgCl_2$, 10 mM PIPES pH 6.8) for 30 sec, in CSK buffer + 0.5% Triton X-100 for 2 min, and again in CSK buffer for 30 sec. Cells were fixed in 4% paraformaldehyde in PBS for 10 min and dehydrated through a series of 70%, 80%, 95%, and 100% EtOH for 2 min each. After airdrying the slides, hybridization was performed by applying probe to the cells. Probes were covered with a coverslip and sealed with rubber cement. Slides were then placed in a humidified chamber and incubated overnight at 37°C. Coverslips were removed and slides were washed in 2X SSC + formamide (50%) for 15 min at 39°C, in 2X SCC three times for 5 min each at 39°C, in 1X SSC for 10 min, and in 4X SSC with a dip (20X SSC: 3 M NaCl, 0.3 M tri-sodium citrate dihydrate in H_2O). Cellular DNA was counterstained by incubating slides in 4X SSC + 0.1% Tween + 14 mM DAPI for 1.5 min. Slides were washed with 4X SSC for 5 min. After washing, the sample was mounted in Vectashield (Vector Laboratories) and covered with a coverslip. Samples were imaged under the microscope (Axio Observer Z1, Zeiss) equipped with an ORCA-Flash4.0 camera (Hamamatsu Photonics K.K.). Images were processed using Zeiss Zen Pro 2.0 software.

X chromosome painting

X chromosome staining procedures were performed after imaging RNA FISH samples. To strip off RNA FISH dye and DAPI staining, the coverslips were removed and the glass slides were washed in 2X SSC + formamide (70%). The flask was incubated in a water bath at

85°C for 10 min. Subsequently the slides were washed three times in 2X SSC at RT for 5 min each. For detecting the mouse X chromosome, XMP X Green probe (MetaSystems Probes, D-1420-050-FI) was 1:3 diluted in Hybrisol VII (MP Biomedicals) and added to the sample. The samples were covered by coverslips, sealed with rubber cement, and put on a heat block at 75°C for 2 min. Then, the slides were placed in a humidified chamber and incubated at 37°C overnight. The next day, cover slips were removed. Glass slides were washed in 0.4X SSC at 72°C for 2 min, followed by 2X SSC, 0.05% Tween at RT for 30 sec, and then counterstained with 14 mM DAPI in 2X SSC, 0.05% Tween at RT for 90 sec. Then, slides were washed in 2X SSC at RT for 5 min. The samples were mounted in Vectashield Antifade Mounting Medium (Vector Laboratories) and covered with cover slips. The samples were imaged under the microscope (Axio Observer Z1, Zeiss) equipped with an ORCA-Flash4.0 camera (Hamamatsu Photonics K.K.). Images were processed using Zeiss Zen Pro 2.0 software.

Supplemental References

Aizawa, E., Dumeau, C.-E., Freimann, R., Di Minin, G., and Wutz, A. (2020a). Polyploidy of semi-cloned embryos generated from parthenogenetic haploid embryonic stem cells. PLoS One *15*, e0233072. 10.1371/journal.pone.0233072.

Aizawa, E., Dumeau, C.E., and Wutz, A. (2020b). Application of Mouse Parthenogenetic Haploid Embryonic Stem Cells as a Substitute of Sperm. J Vis Exp. 10.3791/61999.

He, Z.Q., Xia, B.L., Wang, Y.K., Li, J., Feng, G.H., Zhang, L.L., Li, Y.H., Wan, H.F., Li, T.D., Xu, K., et al. (2017). Generation of Mouse Haploid Somatic Cells by Small Molecules for Genome-wide Genetic Screening. Cell Rep. *20*, 2227-2237. 10.1016/j.celrep.2017.07.081.

Wutz, A., and Jaenisch, R. (2000). A shift from reversible to irreversible X inactivation is triggered during ES cell differentiation. Mol. Cell *5*, 695-705. 10.1016/s1097-2765(00)80248-8.
