# Supplementary material for: Haploid mouse germ cell precursors from embryonic stem cells reveal Xist activation from a single X chromosome
Source: Stem Cell Reports. 2021 Dec 16;17(1):43–52. doi: 10.1016/j.stemcr.2021.11.006 (PMC8758942; doi:10.1016/j.stemcr.2021.11.006)
Supplement: Document S2. Article plus supplemental information [file mmc2.pdf]

Stem Cell Reports
Report
OPEN ACCESS

Haploid mouse germ cell precursors from embryonic stem cells reveal *Xist* activation from a single X chromosome

Eishi Aizawa,[1,2] Corinne Kaufmann,[1] Sarah Sting,[1] Sarah Boigner,[1] Remo Freimann,[1] Giulio Di Minin,[1] and Anton Wutz[1,*]
[1]Institute of Molecular Health Sciences, Swiss Federal Institute of Technology, ETH Zurich, Zurich, Switzerland
[2]Present address: Laboratory for Chromosome Segregation, RIKEN Center for Biosystems Dynamics Research, Kobe, Japan
*Correspondence: awutz@ethz.ch
https://doi.org/10.1016/j.stemcr.2021.11.006

SUMMARY

Mammalian haploid cells have applications for genetic screening and substituting gametic genomes. Here, we characterize a culture system for obtaining haploid primordial germ cell-like cells (PGCLCs) from haploid mouse embryonic stem cells (ESCs). We find that haploid cells show predisposition for PGCLCs, whereas a large fraction of somatic cells becomes diploid. Characterization of the differentiating haploid ESCs (haESCs) reveals that *Xist* is activated from and colocalizes with the single X chromosome. This observation suggests that X chromosome inactivation (XCI) is initiated in haploid cells consistent with a model where autosomal blocking factors set a threshold for X-linked activators. We further find that *Xist* expression is lost at later timepoints in differentiation, which likely reflects the loss of X-linked activators. *In vitro* differentiation of haploid PGCLCs can be a useful approach for future studies of potential X-linked activators of *Xist*.

INTRODUCTION

In mice, the germline is specified from proximal epiblast, and primordial germ cells (PGCs) are segregated from somatic lineages in the embryo. PGC differentiation has been recapitulated in cultures of mouse embryonic stem cells (ESCs), which enabled the generation of functional gametes (Hayashi et al., 2011, 2012). Progress in culture techniques is opening opportunities for studies of the mammalian germline.

Mammalian dosage compensation is facilitated by inactivation of one of the two X chromosomes in female cells (Lyon, 1961). In mice, X chromosome inactivation (XCI) is initiated by the long noncoding *Xist* RNA, which is expressed from, and accumulates over, the inactive X chromosome (Xi) before X-linked gene repression (Galupa and Heard, 2018). In the developing female epiblast, two active X chromosomes (Xas) are present before embryonic day (E) 5.5, when random XCI is initiated (Mak et al., 2004). Thereafter, the Xi is maintained in somatic lineages, but Xi reactivation is observed in the female germline (Sugimoto and Abe, 2007).

The regulation of XCI remains to be fully understood. A number of observations suggest that the X to autosome (X:A) ratio controls the initiation of XCI and *Xist* expression. One model posits that autosomal blocking factors prevent *Xist* activation for explaining the observation that a single X chromosome is insufficient for initiation of XCI in male cells (Barakat et al., 2014; Kay et al., 1993; Pollex and Heard, 2019). In female cells, twice the number of X-linked activators overcomes an activation threshold for *Xist*, leading to the initiation of XCI. An alternative model is based on the observation of pairing the X chromosomes at the XCI center (*Xic*) (Bacher et al., 2006; Xu et al., 2006, 2007). The *Xic* encompasses the *Xist* gene and other regulators of XCI, including the antisense *Tsix* transcript. Genetic elements that are required for *Xic* pairing have been identified and shown to induce XCI when transgenically integrated into autosomes in male ESCs (Augui et al., 2007). Furthermore, stochastic regulation of *Xist* has been proposed from studies of tetraploid ESCs, where a variable number of X chromosomes displayed activation of *Xist* upon entry into differentiation (Monkhorst et al., 2008). Investigating the X-counting mechanism in the context of different autosomal dosage has led to further understanding of the underlying regulation.

The establishment of haploid ESCs (haESCs) has advanced the study of the effects of genome ploidy on cells (Elling et al., 2011; Leeb and Wutz, 2011). HaESCs possess a haploid genome but also show a tendency toward diploidization, which is strongly enhanced when haESCs enter differentiation. Several factors that affect diploidization have been extensively investigated in somatic lineage differentiation, with the aim to reduce the high rates of diploidization (Freimann and Wutz, 2017; He et al., 2017, 2018; Olbrich et al., 2017, 2019; Takahashi et al., 2014).

Here, we report the successful differentiation of haESCs into haploid primordial germ cell-like cells (PGCLCs) *in vitro*. We observed that haploid cells showed predisposition for PGCLCs over somatic cells. We then use this system to investigate *Xist* activation in haploid cells. Our data demonstrate that a single X chromosome is sufficient for *Xist* activation in a haploid genome consistent with a lower threshold of autosomal blocking factors. Although

This is an open access article under the CC BY license (http://creativecommons.org/licenses/by/4.0/).

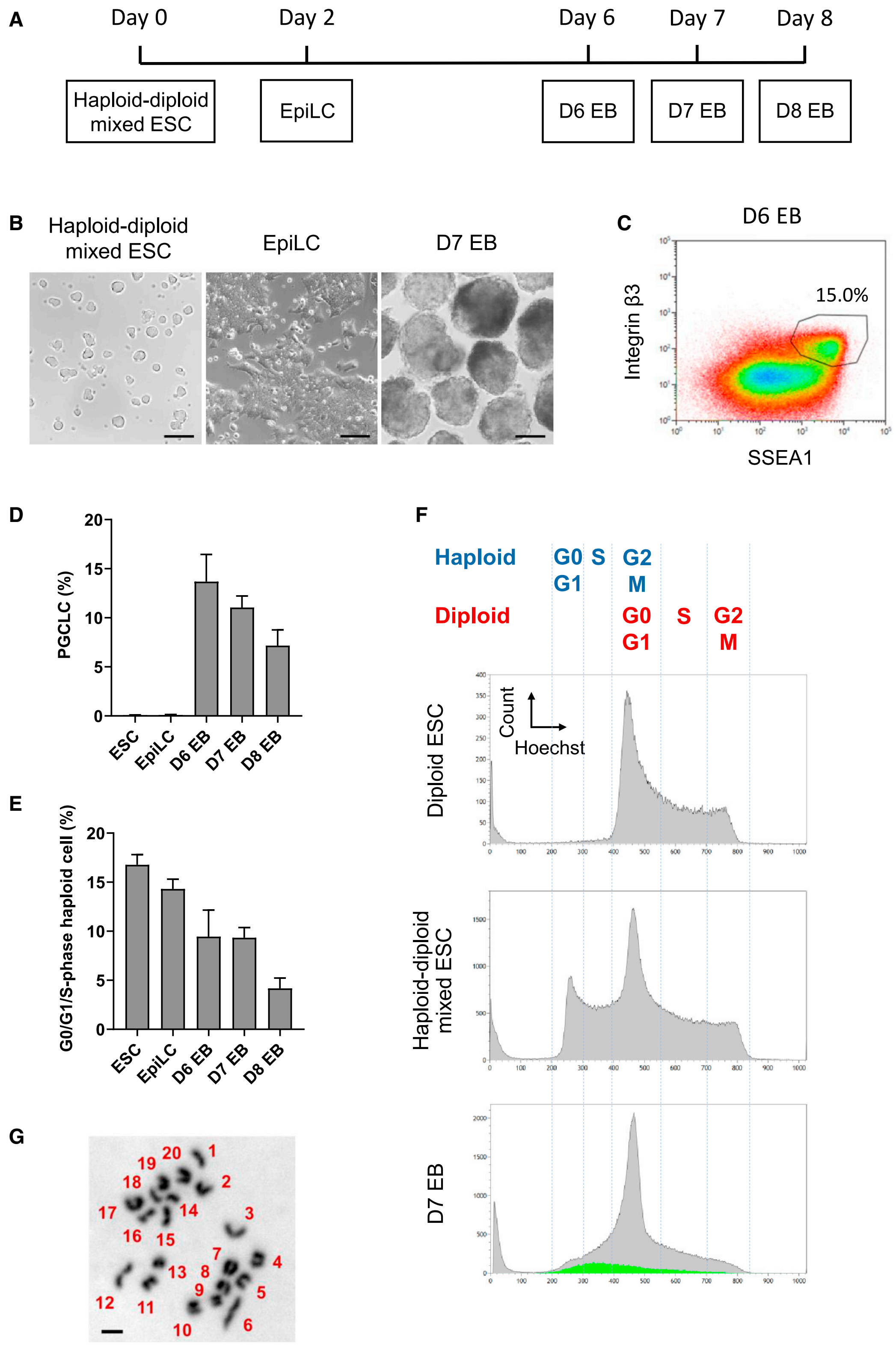

Figure 1. HaESCs differentiate to haploid PGCLCs *in vitro*

(A) A scheme of germ cell differentiation of haploid-diploid-mixed ESCs.

(B) Morphology of ESCs, EpiLCs, and d7 EBs derived from haploid-diploid-mixed ESCs. Scale bar, 100 μm.

(legend continued on next page)

survival of haploid cells is paralleled by a downregulation of *Xist* that can likely be explained by a loss of X-linked activators, we also find that deletion of *Xist* is not sufficient to prevent diploidization of haESCs during neural lineage differentiation.

RESULTS

Differentiation of haESCs into haploid PGCLCs

To investigate the feasibility of germ cell differentiation from haESCs we followed a previously established protocol (Hayashi et al., 2011). A mixed population of haESCs and diploid ESCs, containing 16.8% cells with a 1n DNA content (G0/G1/S-phase haploid), were differentiated to epiblast-like cells (EpiLCs) for 2 days in the presence of FGF2 and Activin A (Figures 1A and 1B). Subsequently, we aggregated the EpiLCs for embryoid body (EB) formation. PGCLCs appeared between days 6 and 8 and were identified by co-expression of the SSEA1 and integrin β3 surface markers (Figures 1C and 1D). Analysis of the ploidy distribution by flow cytometry showed that 9.3% of all cells on day 7 possessed a 1n DNA content corresponding to haploid cells in G0-, G1-,and S-phase, which is approximately half the fraction of haESCs at the beginning of differentiation (16.8%; Figures 1E and 1F). To confirm a haploid karyotype in PGCLCs, we sorted cells expressing SSEA1 and integrin β3 from d7 EBs and prepared chromosome spreads. As expected, a set of 20 acrocentric chromosomes typical for an intact haploid mouse genome could be observed (Figure 1G). Transcription analysis of sorted PGCLCs revealed the upregulation of the PGC markers *Blimp1*, *Prdm14*, *Tfap2c*, and *Stella* and the downregulation of *Dnmt3b* in G0/G1/S-phase haploid (1n) as well as in S/G2/M-phase diploid (4n) PGCLCs relative to EpiLCs and ESCs (Figure S1A). These results show that differentiation of haploid PGCLCs *in vitro* recapitulates gene expression changes that are anticipated from the development of PGCs *in vivo*.

PGCLCs are predisposed to possess a haploid genome

To further analyze the ploidy distribution within the PGCLC population, we used flow cytometry to plot the Hoechst 33,342 intensity of all cells in d7 EBs against the expression of integrin β3 (Figure 2A). The integrin β3 strongly positive PGCLC population appeared to have a notably high content of haploid cells compared with somatic lineages, which were weakly positive or negative for integrin β3. We therefore further characterized the percentage of PGCLCs in windows of different DNA content (Figures 2B–2D and S1B). PGCLCs accounted for 32.3% of 1n (G0/G1/S-phase) haploid cells, while 11.1% of all cells and less than 10% of cell population with higher DNA content expressed both PGC markers on average in 18 experiments (Figures 2B and S1B). Therefore, the 1n haploid population contained a 3-fold higher percentage of PGCLCs. The apparent enrichment of haploid cells within the PGCLC population can in part be attributed to high diploidization rates that accompany differentiation into somatic lineages. Both SSEA1 and integrin β3 positive PGCLC population demonstrated a significantly higher proportion of G0/G1/S haploid cell population than somatic lineages, which were negative for SSEA1 and/or integrin β3 (Figure 2C). Additionally, a substantially higher percentage of haploid cells was observed in PGCLCs (27.5%) compared with the overall population of ESCs (16.8%), EpiLCs (14.3%), and d7 EBs (9.3%; Figure 2D). These results indicate that PGCLCs have a predisposition to possess a haploid genome.

Xist is activated from a single X chromosome in a haploid genome

Germline differentiation facilitates an investigation of *Xist* expression in haploid cells without caveats that arise from diploidization or cell death. We used sorted 1n (G0/G1/S-phase haploid) and 4n (S/G2/M-phase diploid) cells at different timepoints for *Xist* RNA fluorescence *in situ* hybridization (FISH) analysis during germ cell differentiation. One and two punctate *Xist* signals were observed in haESCs and diploid ESCs, respectively (Figure 3A). We used double-stranded probes that recognize not only nascent *Xist* but also *Tsix* transcripts before initiation of XCI as a punctate focal signal. The majority of haESCs and diploid ESCs showed no *Xist* cluster (Figure 3B). After the initiation of differentiation, *Xist* RNA clusters were observed in 42.0% and 59.0% of haploid and diploid EpiLCs, respectively. *Xist* is activated from the single X chromosome in haploid cells with a similar frequency as from one of the two X chromosomes in diploid cells. Strong upregulation of *Xist* was also confirmed by quantitative RT-PCR analysis in

(C) A representative flow cytometry analysis of d6 EBs derived from haploid-diploid-mixed ESCs. PGCLCs, positive for both SSEA1 and integrin β3, accounted for 15.0% out of all cells.

(D and E) The proportion of PGCLCs (D) and G0/G1/S-phase haploid cells (E) out of all cells at the different stages during germ cell differentiation of haploid-diploid-mixed ESCs. Data are derived from 14 (ESC), 9 (EpiLC), 7 (d6 EB), 18 (d7 EB), and 7 (d8 EB) independent experiments. The data represent the mean value and the standard error of the mean.

(F) Flow cytometry analysis of DNA content of diploid ESCs, haploid-diploid-mixed ESCs, and d7 EBs derived from haploid-diploid-mixed ESCs. Cell cycle phases of haploid and diploid cells (top), and PGCLCs in d7 EBs (green) are indicated.

(G) Representative chromosome spread of a haploid d7 PGCLC. Scale bar, 5 μm.

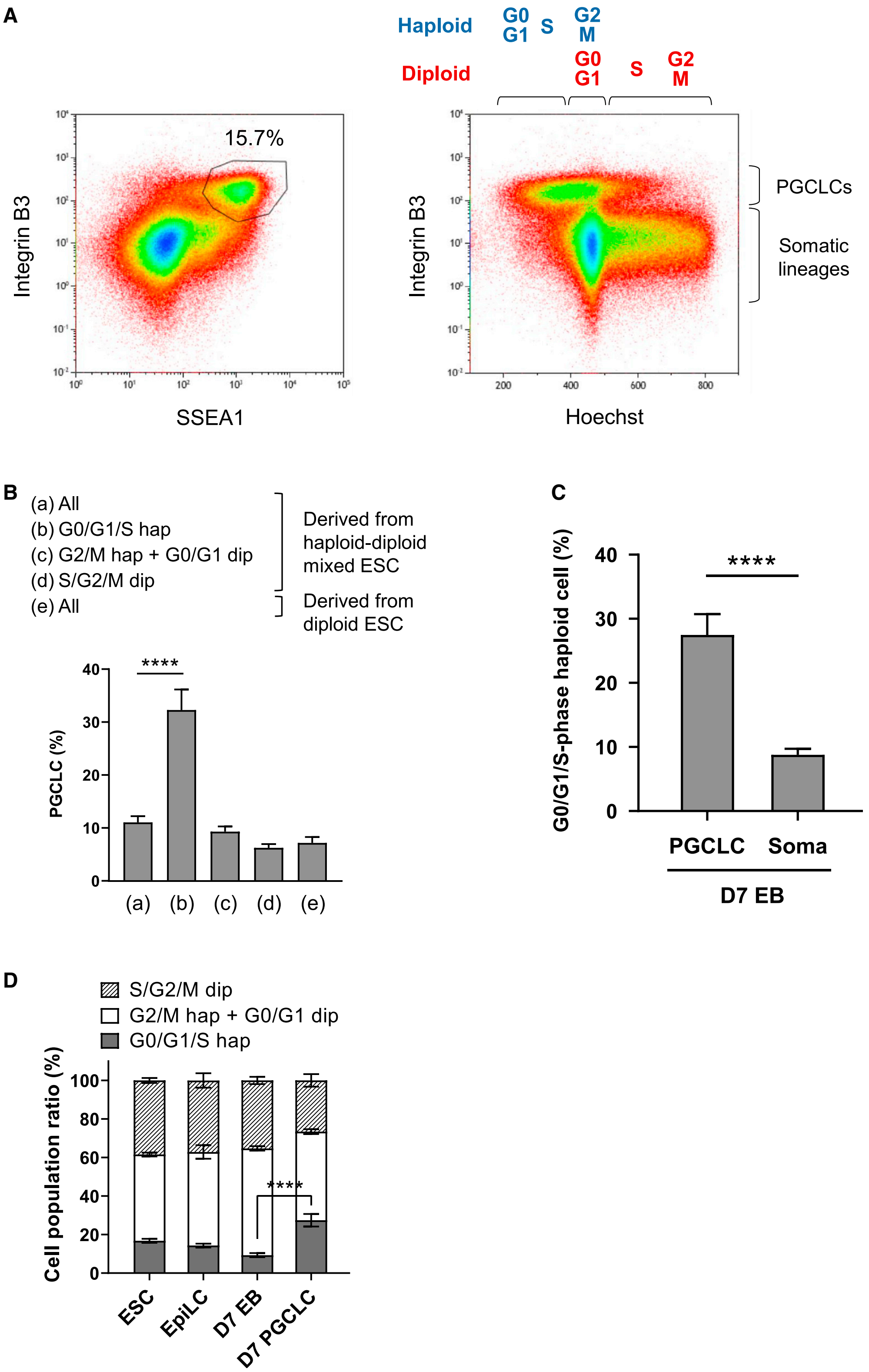

(legend on next page)

both haploid and diploid EpiLCs (Figure S1A). With further differentiation, only 8.7% and 24.0% of haploid and diploid d7 PGCLCs showed *Xist* clusters, respectively. This observation might reflect the repression of *Xist* in germ cell differentiation and cell selection that is expected from the loss of X-linked gene expression in haploid cells after inactivation of the single X chromosome.

To confirm *Xist* expression in haploid cells and to explore its kinetics, we performed time course analysis of *Xist* RNA FISH combined with subsequent X chromosome painting at 24, 48, and 72 h of EpiLC differentiation (Figures 3C and 3D). As expected, X chromosome painting demonstrated one or two signals in each cell nucleus, which represent haploid and diploid cells, respectively (Figure 3C). We detected *Xist* clusters in 33.7% of haploid cells at 24 h of EpiLC differentiation. The proportion of cells with *Xist* clusters profoundly decreased to 24.2% and 4.6% at 48 and 72 h, respectively. In contrast, diploid cells exhibited a higher proportion of *Xist* clusters at every time point (53.8% at 24 h; 54.7% at 48 h; 33.7% at 72 h). These results indicate that *Xist* expression in haploid cells is transient, and haploid cells preferably repress *Xist* expression compared with diploid cells. We further hypothesize that repression of *Xist* expression contributes to the maintenance of haploid cells, as loss of X-linked gene expression through XCI is incompatible with cell survival.

Mutation of *Xist* is insufficient to prevent diploidization of haESCs

To further explore if *Xist* repression contributes to the maintenance of haploidy, haESC lines deficient in the *Xist* gene were established (Figures 4A–4D and S2). We engineered a deletion within *Xist* exon 1 using paired RNA guided nuclease vectors (Figure 4A). Three *ΔXist* haESC clones that carried a deletion of about 6,380 bp were established (Figures 4B–4D). Silencing of *Xist* expression in a *ΔXist* ESC line was also confirmed by RNA FISH after EpiLC differentiation for 48 h (Figure S2).

We analyzed neural differentiation as a representative of somatic lineage differentiation. Using the *ΔXist* haESC line, differentiation of haploid-diploid-mixed ESCs into neural stem cell-like cells (NSCLCs) was performed by following a published protocol (He et al., 2017) (Figure 4E). Treatment with ROCKi was applied to repress diploidization of haploid cells in neural differentiation. Under these conditions, parental wild-type (WT) ESCs maintained a G0/G1/S-phase haploid cell population at the ratio from 36.7% of ESCs to 11.5% of NSCLC at day 7 in differentiation (Figure 4F). Similarly, *ΔXist* ESCs showed a reduction in the G0/G1/S-phase haploid cell population during the differentiation to NSCLCs. Statistical analysis indicated no significant difference in the loss of the G0/G1/S-phase haploid cell proportion during the differentiation between WT and *ΔXist* haploid-diploid-mixed ESCs (Figure 4G). From these data, we conclude that the mutation of *Xist* is not sufficient to prevent diploidization of haESCs during somatic lineage differentiation. Further studies are required to understand why germ cell precursors have a predisposition to haploidy compared with somatic lineages.

DISCUSSION

Our observation that haESCs maintain a haploid genome during germ cell differentiation enabled us to analyze *Xist* activation in the context of a haploid genome. Our results are explained by the idea that the amount of blocking factors produced from a single set of autosomes is insufficient to counteract activators from a single X chromosome. In contrast, X-linked activators are titrated by a double dose of blocking factors in diploid male cells preventing *Xist* activation. Our result therefore supports a model of diffusible X-linked activators and autosomal blocking factors (Barakat et al., 2014; Pollex and Heard, 2019). Previous studies have also linked the activation of *Xist* with *Xic* pairing in differentiating diploid ESCs (Bacher et al., 2006; Xu et al., 2006). *Xic* pairing cannot occur in haploid cells, as

Figure 2. Predisposition of haploid cells for PGCLCs over somatic lineage differentiation

(A) A representative flow cytometry analysis of d7 EBs derived from haploid-diploid-mixed ESCs. PGCLCs, positive for both SSEA1 and integrin β3, accounted for 15.7% out of all cells (left). The cell type and DNA content analyzed by the dye intensity of integrin β3 and Hoechst 33,342, respectively, are shown (right). The cell cycle profile of haploid and diploid cells is shown at the top.

(B) The ratio of PGCLCs in cell populations of different DNA content in d7 EBs derived from haploid-diploid-mixed ESCs or diploid ESCs. PGCLCs accounted on average for 32.3% out of G0/G1/S haploid cells in d7 EBs derived from haploid-diploid-mixed ESCs. Data represent the mean and the standard error of the mean. The data are derived from 18 (d7 EBs derived from haploid-diploid-mixed ESCs) and 12 (d7 EBs derived from diploid ESCs) individual experiments.

(C) The proportion of G0/G1/S-phase haploid cell population of PGCLCs (positive for both SSEA1 and integrin β3) and somatic lineages (negative for SSEA1 and/or integrin β3) in d7 EBs derived from haploid-diploid-mixed ESCs. The data are derived from 18 individual experiments.

(D) Cell cycle distribution of haploid and diploid cells during germ cell differentiation of haploid-diploid-mixed ESCs. Data represent the mean value and the standard error of the mean. The data are derived from 14 (ESC), 9 (EpiLC), and 18 (d7 EB and d7 PGCLC) individual experiments. ****$p < 0.0001$.

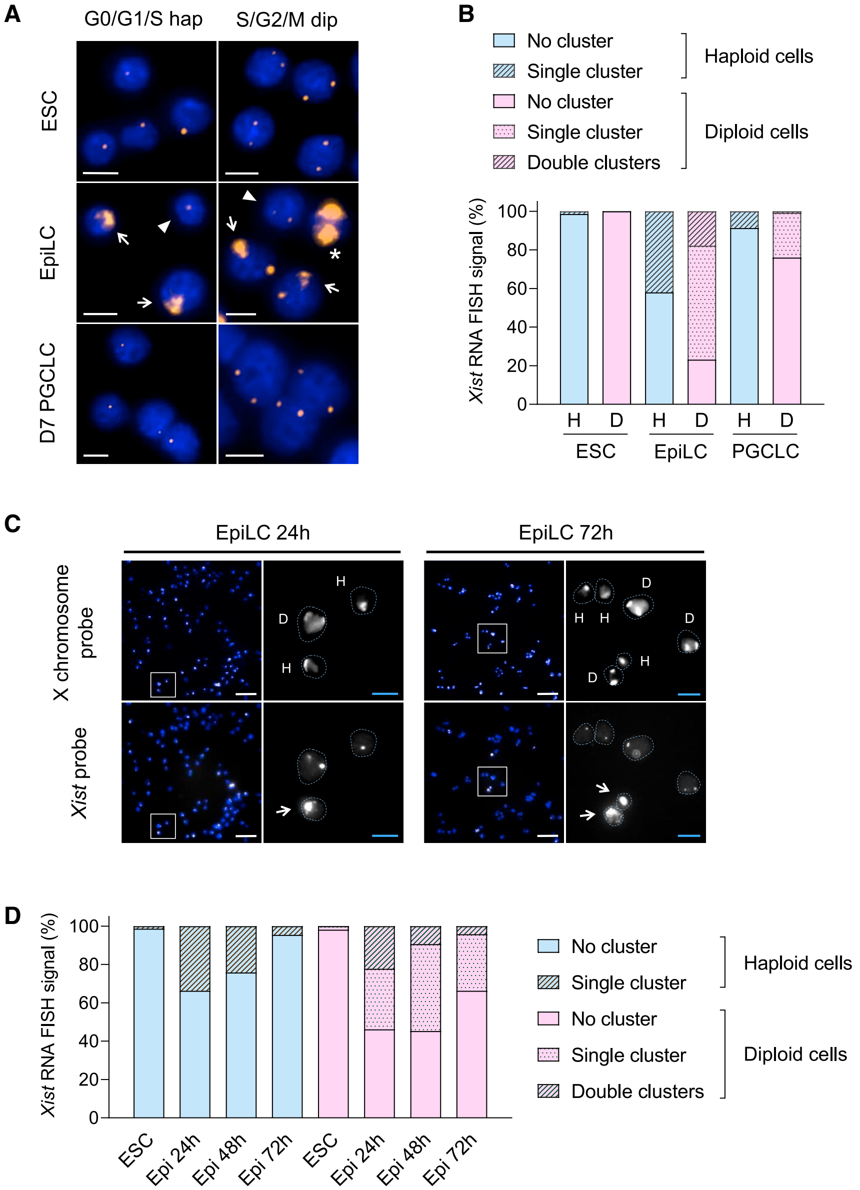

Figure 3. *Xist* is activated and repressed in both haploid and diploid cells during EpiLC and PGCLC differentiation

(A) Representative images of *Xist* expression during germ cell differentiation of haploid-diploid-mixed ESCs detected by RNA FISH using a Cy3-labeled *Xist* probe (orange). No *Xist* cluster but single foci are observed in G0/G1/S-phase haESCs and PGCLCs. No cluster but double foci are observed in S/G2/M-phase diploid ESCs and PGCLCs. Single *Xist* clusters (arrow) and foci (arrowhead) observed in G0/G1/S-phase haploid EpiLCs. Double clusters (asterisk), single clusters (arrow), and no cluster (arrowhead) observed in S/G2/M-phase diploid EpiLC. Scale bar, 10 μm.

(B) Proportion of *Xist* RNA FISH signals during germ cell differentiation of haploid-diploid-mixed ESCs. The number of cells possessing no, single, or double *Xist* RNA clusters were counted in G0/G1/S-phase haploid and S/G2/M diploid cells. Total numbers of counted cells were 138 (H, ESC), 171 (D, ESC), 100 (H, EpiLC), 173 (D, EpiLC), 69 (H, PGCLC), and 121 (D, PGCLC) derived from 2 independent experiments for each sample. D, S/G2/M-phase diploid; H, G0/G1/S-phase haploid.

(C) Representative images of haploid-diploid-mixed ESCs during EpiLC differentiation for 24 and 72 h. X chromosome painting and *Xist* expression were detected by DNA FISH using a X-chromosome-specific probe (white) and RNA FISH using a *Xist* probe (white), respectively. Nuclei are shown in blue (DAPI staining). The area marked by white squares in the left images is enlarged on the right. Nuclei are delineated with cyan dashed lines in enlarged images. Haploidy (H) or diploidy (D) of cells are indicated based on the number of X chromosome detected. Arrows indicate *Xist* clusters. Scale bar (white), 50 μm; scale bar (cyan), 10 μm.

(D) Proportion of *Xist* RNA FISH signals during EpiLC differentiation of haploid-diploid-mixed ESCs for 72 h. The number of cells possessing no, single, or double *Xist* RNA clusters were counted in haploid and diploid cells, in which ploidies were identified by X chromosome painting. Total numbers of counted cells were 223 (haploid, ESC), 261 (haploid, EpiLC 24h), 285 (haploid, EpiLC 48h), 196 (haploid, EpiLC 72h), 105 (diploid, ESC), 117 (diploid, EpiLC 24h), 128 (diploid, EpiLC 48h), and 95 (diploid, EpiLC 72h). Epi, EpiLC.

only a single X chromosome is present. Our experiment shows that *Xist* activation does not strictly depend on *Xic* pairing. This observation does not rule out that *Xic* pairing contributes to XCI in diploid cells or has a role in ensuring that one X chromosome remains active after the decision for initiating XCI has been taken in female cells. A recent study has reported on engineering the *Xic* regions for tethering to the nuclear lamina (Pollex and Heard, 2019). XCI was initiated in female mouse ESCs, despite *Xic* movement being restricted.

Our study also reveals that PGCLCs have a remarkable predisposition to a haploid genome. PGCs have a similar epigenetic state to ESCs, which might contribute to tolerance of a haploid genome. Firstly, during migration to the gonads, the Xi becomes reactivated, suggesting that dosage compensation is not essential for germ cell development. Secondly, ESCs and PGCs exhibit genome-wide DNA hypomethylation (Leitch et al., 2013). Lastly, transcription factors that are associated with pluripotent cells and germ cells including *Oct4* have been implicated as repressors of

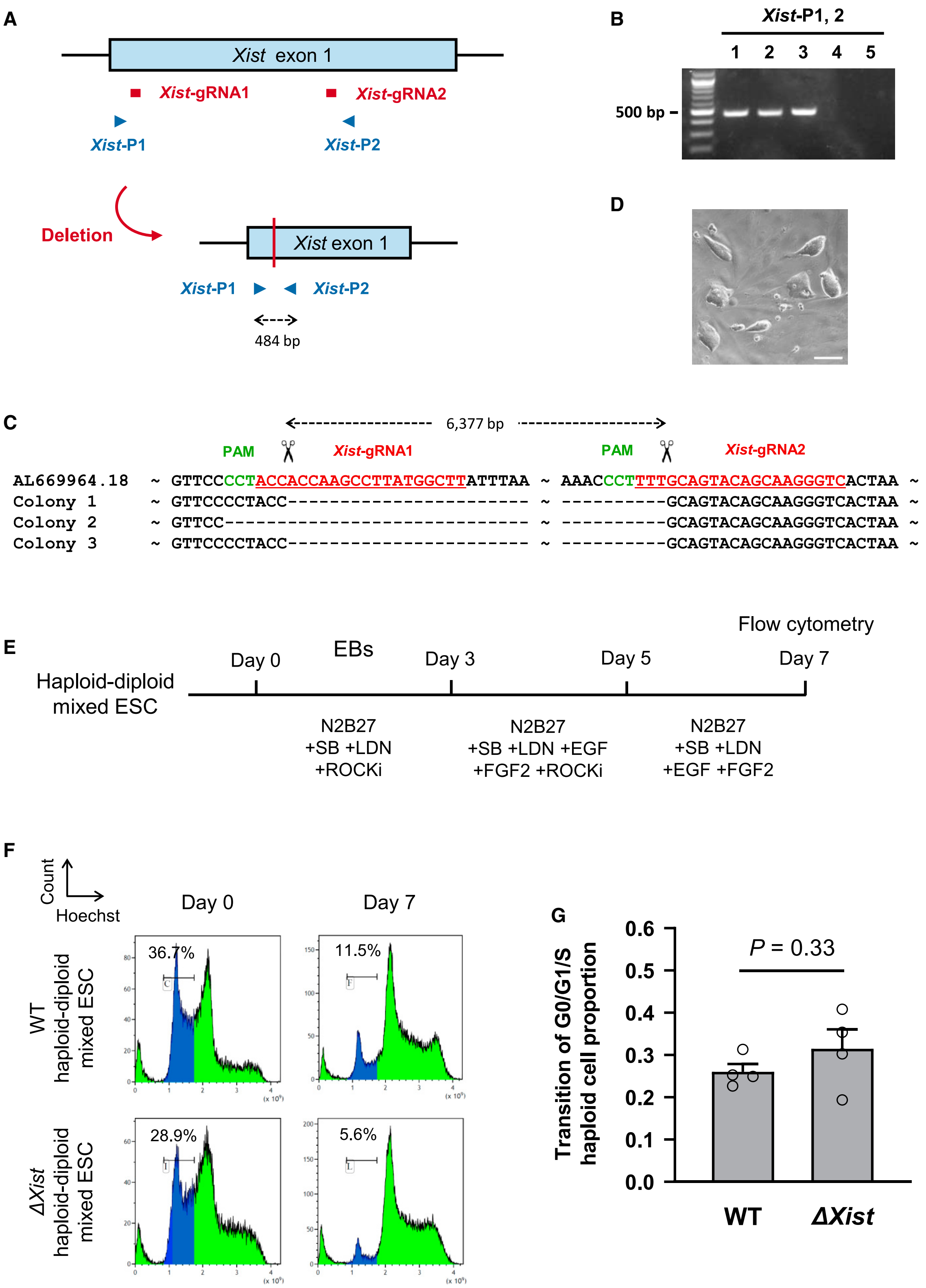

Figure 4. Effect of *Xist* deletion on haploid maintenance during neural lineage differentiation of haESCs

(A) Design of gRNAs and primers for targeting a deletion of *Xist* exon 1.

(B) PCR analysis of 5 ESC clones using primers *Xist*-P1 and *Xist*-P2 identifying deletion of *Xist* exon 1 in 3 haESC lines (1–3).

(legend continued on next page)

Xist (Donohoe et al., 2009; Navarro et al., 2010; Nesterova et al., 2011). It is conceivable that the expression of potential repressors of *Xist* in PGCs might contribute to the maintenance of a haploid genome consistent with our finding that *Xist* expression is lost at later time points in differentiating cultures. Although *Oct4* is also expressed in the epiblast, other factors have also been implicated in *Xist* repression in pluripotent cells. We have tested the relevance of *Xist* for diploidization of NSCLCs and find that the deficiency of *Xist* does not improve the maintenance of a haploid genome. *Xist* is repressed in haploid cells during the EpiLC differentiation, whereas it comparatively persists in diploid cells (Figure 3D). This suggests a mechanism of *Xist* repression in differentiation. Since autosomal blocking factors are lost during differentiation, we propose that the downregulation of *Xist* is a consequence of the loss of X-linked activators. Conceivably, X-linked activators of *Xist* are dosage sensitive and therefore could possess a higher turnover rate than other X-linked genes that are required for cell survival. Haploid PGCLCs will be useful for future studies of ploidy restriction and for genetic exploration of potential X-linked activators of *Xist*.

EXPERIMENTAL PROCEDURES

Derivation and culture of a haESC line

All animal experiments were performed under the license ZH152/17 in accordance with the standards and regulations of the Cantonal Ethics Commission Zurich. Derivation of a haESC line from 129S6/SvEvTac mice was performed as previously described (Leeb and Wutz, 2011). At passage 5, a G0/G1/S-phase haploid cell population of the haESC line was purified by fluorescence-activated cell sorting (FACS) using a flow cytometer (MoFlo Astrios EQ, Beckman Coulter) after Hoechst 33,342 (Invitrogen) staining. Sorted cells were cultured and maintained on a gelatin-coated plate with irradiated mouse embryonic fibroblasts (MEFs) from E12.5 DR4 mouse embryos (The Jackson Laboratory, no. 003208). Cells were maintained in Serum + 2i + LIF medium, which was prepared by mixing equal volumes of Serum + LIF medium without 2i (Postlmayr et al., 2020) and 2i + LIF medium (Hayashi and Saitou, 2013). At passage 10, the G0/G1/S-phase haploid cell population of the haESC line was purified by FACS and maintained on a gelatin-coated plate with MEFs in Serum + 2i + LIF medium.

In vitro germ cell differentiation

Germ cell differentiation was performed following a published protocol (Hayashi and Saitou, 2013) with a few modifications. The haESC line was cultured on an ornithine- and laminin-coated plate without MEFs in 2i + LIF medium from passage 12. At passage 15, EpiLC differentiation was initiated as described in the protocol. After 48 h of EpiLC differentiation, 2.3 x 10^5 EpiLCs were plated into a well of a Sphericalplate 5D (Kugelmeiers Ltd.) with 1.4 mL of PGCLC differentiation medium without BMP8a. After 4 days of PGCLC differentiation, half of the medium was replaced with fresh PGCLC differentiation medium without BMP4 and BMP8a.

For analysis of *Xist* RNA FISH together with X chromosome painting, the haESC line was subjected to EpiLC differentiation as described in the protocol (Hayashi and Saitou, 2013). The cells were cultured for a total of 72 h by changing all the medium of EpiLC differentiation every single day.

Flow cytometry analysis and cell sorting

To investigate the cell cycle of haploid and diploid cells and PGCLCs, flow cytometry analysis of ESCs, EpiLCs, and EBs was performed by the following procedures. Cells were harvested from culture vessels as described in a published protocol (Hayashi and Saitou, 2013), followed by staining with 15 μg/mL Hoechst 33,342 for 12 min at 37°C. Subsequently, PE anti-integrin β3 (BioLegend, no. 104307) and eFluor 660 anti-SSEA1 (eBioscience, no. 50881341) were added to the cell suspension at concentration of 1 μg/mL and 0.12 μg/mL, respectively, and the cell sample was kept on ice for 12 min. The fluorescence of each dye was measured by flow cytometry. Cell cycle of haploid and diploid cells was determined based on peaks of the cell population at 1n and 2n DNA contents, corresponding to G0/G1-phase haploid cells and G2/M-phase haploid and G0/G1-phase diploid cells, respectively, by measuring the signal of Hoechst 33,342. The population of PGCLCs was determined by measuring the signals of PE and eFluor 660.

Statistical analysis

For comparison of the ratio of cell population, measurements were analyzed with the GraphPad Prism 8 software using an unpaired t test with Welch's correction. A $p < 0.05$ was considered statistically significant.

SUPPLEMENTAL INFORMATION

Supplemental information can be found online at https://doi.org/10.1016/j.stemcr.2021.11.006.

(C) Sequences of PCR fragments amplified over the deleted region confirmed the loss of targeted *Xist* exon 1 in 3 haESC lines, termed Δ*Xist* ESC lines. The WT sequence is shown on top with PAM sequences and gRNAs indicated.
(D) Morphology of a Δ*Xist* ESC line. Scale bar, 100 μm.
(E) A scheme of NSCLC differentiation of haploid-diploid-mixed ESCs.
(F) Flow cytometry analysis of DNA content on day 0 and 7 during NSCLC differentiation of WT and Δ*Xist* haploid-diploid-mixed ESCs. The population of G0/G1/S-phase haploid cells is indicated in blue color, and its proportion is shown numerically.
(G) Transition of G0/G1/S haploid cell population of WT and Δ*Xist* haploid-diploid-mixed ESCs during NSCLC differentiation. To calculate the transition of G0/G1/S haploid cell population during NSCLC differentiation (Y), the proportion of G0/G1/S haploid cell population on day 0 (X_0) and 7 (X_7) during NSCLC differentiation was applied to the following formula: $Y = X_7/X_0$. Data represent the mean value and the standard error of the mean. The data are derived from 4 experiments.

AUTHOR CONTRIBUTIONS

E.A. and A.W. conceptualized experiments. E.A., C.K., S.S., S.B., R.F., and G.D.M. collected the data. E.A., C.K., and G.D.M. analyzed the data. E.A. and A.W. wrote the manuscript.

CONFLICT OF INTERESTS

The authors declare no competing interests. A.W. is an inventor and owner of patents on mammalian haploid ESCs (European patent 2681310; USA patent 9957479 and 11085020).

ACKNOWLEDGMENTS

This study was supported by the Swiss National Science Foundation (grant 31003A_152814/1).

Received: January 5, 2021
Revised: November 15, 2021
Accepted: November 15, 2021
Published: December 16, 2021

REFERENCES

Augui, S., Filion, G.J., Huart, S., Nora, E., Guggiari, M., Maresca, M., Stewart, A.F., and Heard, E. (2007). Sensing X chromosome pairs before X inactivation via a novel X-pairing region of the Xic. Science *318*, 1632–1636. https://doi.org/10.1126/science.1149420.

Bacher, C.P., Guggiari, M., Brors, B., Augui, S., Clerc, P., Avner, P., Eils, R., and Heard, E. (2006). Transient colocalization of X-inactivation centres accompanies the initiation of X inactivation. Nat. Cell Biol. *8*, 293–299. https://doi.org/10.1038/ncb1365.

Barakat, T.S., Loos, F., van Staveren, S., Myronova, E., Ghazvini, M., Grootegoed, J.A., and Gribnau, J. (2014). The trans-activator RNF12 and cis-acting elements effectuate X chromosome inactivation independent of X-pairing. Mol. Cell *53*, 965–978. https://doi.org/10.1016/j.molcel.2014.02.006.

Donohoe, M.E., Silva, S.S., Pinter, S.F., Xu, N., and Lee, J.T. (2009). The pluripotency factor Oct4 interacts with Ctcf and also controls X-chromosome pairing and counting. Nature *460*, 128–132. https://doi.org/10.1038/nature08098.

Elling, U., Taubenschmid, J., Wirnsberger, G., O'Malley, R., Demers, S.P., Vanhaelen, Q., Shukalyuk, A.I., Schmauss, G., Schramek, D., Schnuetgen, F., et al. (2011). Forward and reverse genetics through derivation of haploid mouse embryonic stem cells. Cell Stem Cell *9*, 563–574. https://doi.org/10.1016/j.stem.2011.10.012.

Freimann, R., and Wutz, A. (2017). A fast and efficient size separation method for haploid embryonic stem cells. Biomicrofluidics *11*, 054117. https://doi.org/10.1063/1.5006326.

Galupa, R., and Heard, E. (2018). X-chromosome inactivation: a crossroads between chromosome architecture and gene regulation. Annu. Rev. Genet. *52*, 535–566. https://doi.org/10.1146/annurev-genet-120116-024611.

Hayashi, K., Ogushi, S., Kurimoto, K., Shimamoto, S., Ohta, H., and Saitou, M. (2012). Offspring from oocytes derived from in vitro primordial germ cell-like cells in mice. Science *338*, 971–975. https://doi.org/10.1126/science.1226889.

Hayashi, K., Ohta, H., Kurimoto, K., Aramaki, S., and Saitou, M. (2011). Reconstitution of the mouse germ cell specification pathway in culture by pluripotent stem cells. Cell *146*, 519–532. https://doi.org/10.1016/j.cell.2011.06.052.

Hayashi, K., and Saitou, M. (2013). Generation of eggs from mouse embryonic stem cells and induced pluripotent stem cells. Nat. Protoc. *8*, 1513–1524. https://doi.org/10.1038/nprot.2013.090.

He, W., Zhang, X., Zhang, Y., Zheng, W., Xiong, Z., Hu, X., Wang, M., Zhang, L., Zhao, K., Qiao, Z., et al. (2018). Reduced self-diploidization and improved survival of semi-cloned mice produced from androgenetic haploid embryonic stem cells through overexpression of Dnmt3b. Stem Cell Rep. *10*, 477–493. https://doi.org/10.1016/j.stemcr.2017.12.024.

He, Z.Q., Xia, B.L., Wang, Y.K., Li, J., Feng, G.H., Zhang, L.L., Li, Y.H., Wan, H.F., Li, T.D., Xu, K., et al. (2017). Generation of mouse haploid somatic cells by small molecules for genome-wide genetic screening. Cell Rep. *20*, 2227–2237. https://doi.org/10.1016/j.celrep.2017.07.081.

Kay, G.F., Penny, G.D., Patel, D., Ashworth, A., Brockdorff, N., and Rastan, S. (1993). Expression of Xist during mouse development suggests a role in the initiation of X chromosome inactivation. Cell *72*, 171–182. https://doi.org/10.1016/0092-8674(93)90658-d.

Leeb, M., and Wutz, A. (2011). Derivation of haploid embryonic stem cells from mouse embryos. Nature *479*, 131–134. https://doi.org/10.1038/nature10448.

Leitch, H.G., McEwen, K.R., Turp, A., Encheva, V., Carroll, T., Grabole, N., Mansfield, W., Nashun, B., Knezovich, J.G., Smith, A., et al. (2013). Naive pluripotency is associated with global DNA hypomethylation. Nat. Struct. Mol. Biol. *20*, 311–316. https://doi.org/10.1038/nsmb.2510.

Lyon, M.F. (1961). Gene action in the X-chromosome of the mouse (Mus musculus L.). Nature *190*, 372–373. https://doi.org/10.1038/190372a0.

Mak, W., Nesterova, T.B., de Napoles, M., Appanah, R., Yamanaka, S., Otte, A.P., and Brockdorff, N. (2004). Reactivation of the paternal X chromosome in early mouse embryos. Science *303*, 666–669. https://doi.org/10.1126/science.1092674.

Monkhorst, K., Jonkers, I., Rentmeester, E., Grosveld, F., and Gribnau, J. (2008). X inactivation counting and choice is a stochastic process: evidence for involvement of an X-linked activator. Cell *132*, 410–421. https://doi.org/10.1016/j.cell.2007.12.036.

Navarro, P., Oldfield, A., Legoupi, J., Festuccia, N., Dubois, A., Attia, M., Schoorlemmer, J., Rougeulle, C., Chambers, I., and Avner, P. (2010). Molecular coupling of Tsix regulation and pluripotency. Nature *468*, 457–460. https://doi.org/10.1038/nature09496.

Nesterova, T.B., Senner, C.E., Schneider, J., Alcayna-Stevens, T., Tattermusch, A., Hemberger, M., and Brockdorff, N. (2011). Pluripotency factor binding and Tsix expression act synergistically to repress Xist in undifferentiated embryonic stem cells. Epigenetics Chromatin *4*, 17. https://doi.org/10.1186/1756-8935-4-17.

Olbrich, T., Mayor-Ruiz, C., Vega-Sendino, M., Gomez, C., Ortega, S., Ruiz, S., and Fernandez-Capetillo, O. (2017). A p53-dependent response limits the viability of mammalian haploid cells. Proc. Natl. Acad. Sci. U S A *114*, 9367–9372. https://doi.org/10.1073/pnas.1705133114.

Olbrich, T., Vega-Sendino, M., Murga, M., de Carcer, G., Malumbres, M., Ortega, S., Ruiz, S., and Fernandez-Capetillo, O. (2019). A chemical screen identifies compounds capable of selecting for haploidy in mammalian cells. Cell Rep. *28*, 597–604.e594. https://doi.org/10.1016/j.celrep.2019.06.060.

Pollex, T., and Heard, E. (2019). Nuclear positioning and pairing of X-chromosome inactivation centers are not primary determinants during initiation of random X-inactivation. Nat. Genet. *51*, 285–295. https://doi.org/10.1038/s41588-018-0305-7.

Postlmayr, A., Dumeau, C.E., and Wutz, A. (2020). Cdk8 is required for establishment of H3K27me3 and gene repression by Xist and mouse development. Development *147*. https://doi.org/10.1242/dev.175141.

Sugimoto, M., and Abe, K. (2007). X chromosome reactivation initiates in nascent primordial germ cells in mice. PLoS Genet. *3*, e116. https://doi.org/10.1371/journal.pgen.0030116.

Takahashi, S., Lee, J., Kohda, T., Matsuzawa, A., Kawasumi, M., Kanai-Azuma, M., Kaneko-Ishino, T., and Ishino, F. (2014). Induction of the G2/M transition stabilizes haploid embryonic stem cells. Development *141*, 3842–3847. https://doi.org/10.1242/dev.110726.

Xu, N., Donohoe, M.E., Silva, S.S., and Lee, J.T. (2007). Evidence that homologous X-chromosome pairing requires transcription and Ctcf protein. Nat. Genet. *39*, 1390–1396. https://doi.org/10.1038/ng.2007.5.

Xu, N., Tsai, C.L., and Lee, J.T. (2006). Transient homologous chromosome pairing marks the onset of X inactivation. Science *311*, 1149–1152. https://doi.org/10.1126/science.1122984.

Stem Cell Reports, Volume *17*

Supplemental Information

Haploid mouse germ cell precursors from embryonic stem cells reveal *Xist* activation from a single X chromosome

Eishi Aizawa, Corinne Kaufmann, Sarah Sting, Sarah Boigner, Remo Freimann, Giulio Di Minin, and Anton Wutz

Figure S1

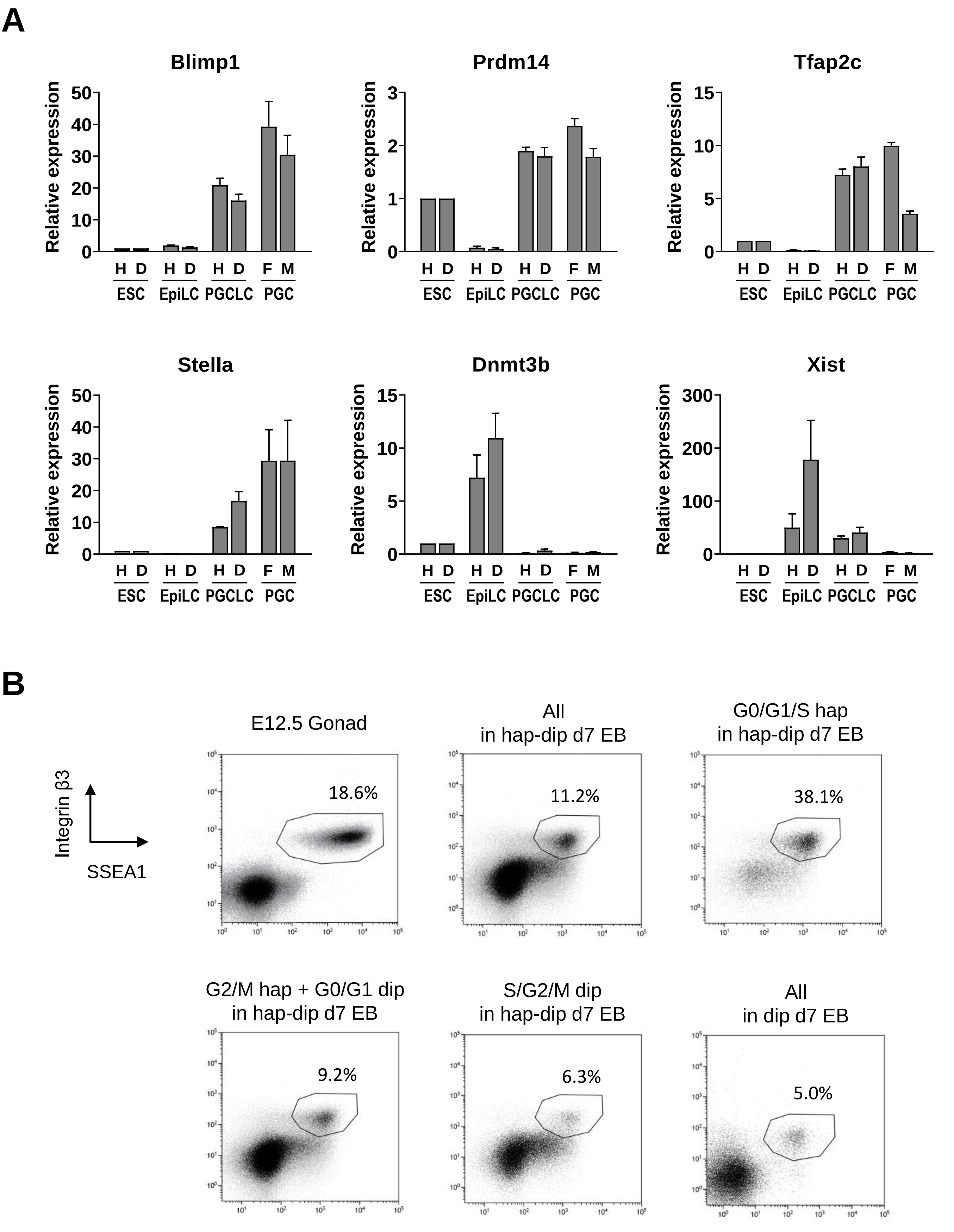
A
Blimp1
Prdm14
Tfap2c
Stella
Dnmt3b
Xist
Relative expression
H D H D H D F M
ESC EpiLC PGCLC PGC
B
Integrin β3
SSEA1
E12.5 Gonad
18.6%
All
in hap-dip d7 EB
11.2%
G0/G1/S hap
in hap-dip d7 EB
38.1%
G2/M hap + G0/G1 dip
in hap-dip d7 EB
9.2%
S/G2/M dip
in hap-dip d7 EB
6.3%
All
in dip d7 EB
5.0%

Figure S2

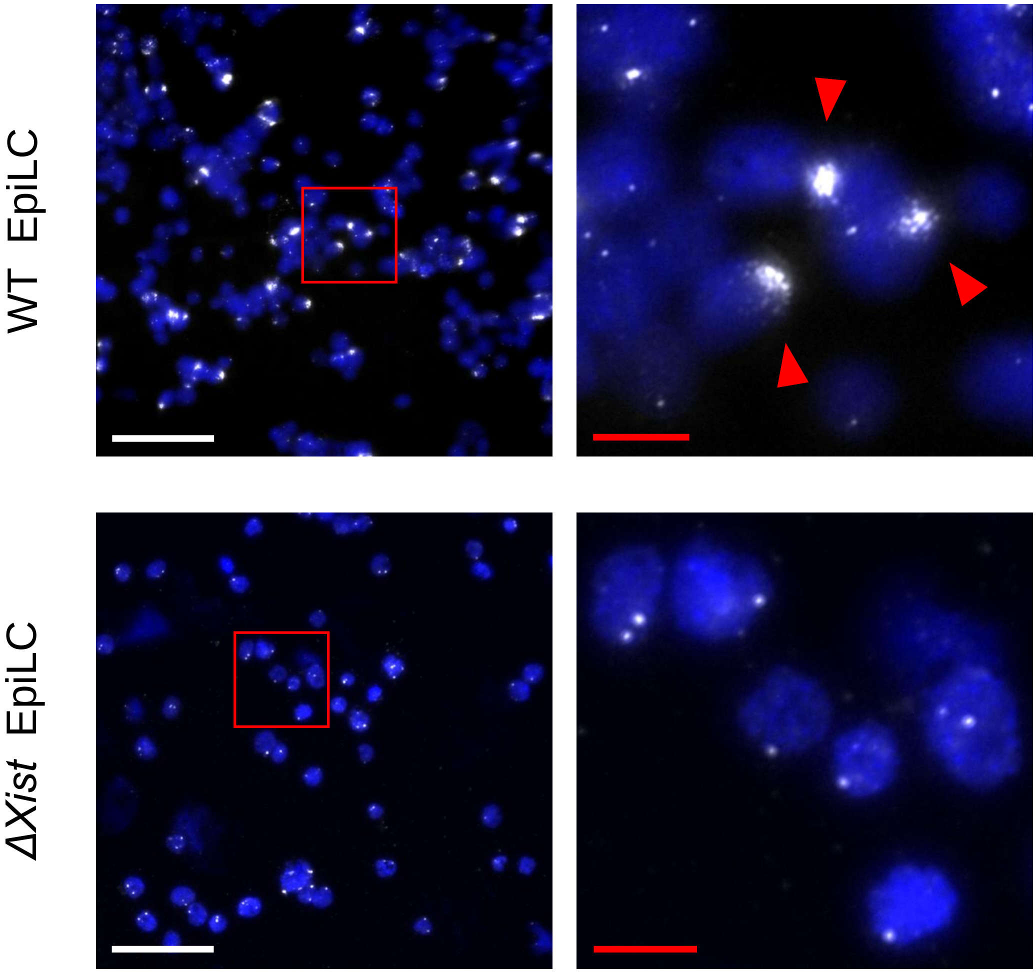

Supplemental Figure Legends

Figure S1. Transcription profile and flow cytometry analysis during germ cell differentiation *in vitro*, related to Figures 1-3. (A) Transcription of PGC markers (*Blimp1*, *Prdm14*, *Tfap2c* and *Stella*), *Dnmt3b* and *Xist* during germ cell differentiation of haploid-diploid mixed ESCs. Gene expression of G0/G1/S-phase haploid and S/G2/M-phase diploid cells of ESCs, EpiLCs and d7 EBs was measured, respectively. Female and male PGCs purified from E12.5 gonads by FACS were used as controls. Gene expression of G0/G1/S-phase haploid and S/G2/M-phase diploid samples was normalized to *Gapdh* expression relative to G0/G1/S-phase haploid and S/G2/M-phase diploid ESCs, respectively. Gene expression of male and female PGCs was normalized to *Gapdh* expression relative to S/G2/M-phase diploid ESCs. Data are derived from 4 biological replicates of each sample. The data represents relative expression with the mean value and the standard error of the mean. H, G0/G1/S-phase haploid; D, S/G2/M-phase diploid; F, female; M, male. (B) A representative flow cytometry analysis of d7 EBs. PGCLCs accounted for 11.2% out of all cells in d7 EBs derived from haploid-diploid mixed ESCs. PGCLCs accounted for 38.1%, 9.2% and 6.3% out of G0/G1/S haploid cell population, G2/M haploid + G0/G1 diploid cell population, and S/G2/M diploid cell population of d7 EBs derived from haploid-diploid mixed ESCs, respectively. Embryonic female gonads at E12.5 and d7 EBs derived from diploid ESCs were also analyzed as controls.

Figure S2. Effect of *Xist* deletion on RNA FISH using a *Xist* probe, related to Figure 4. Representative images of WT and *ΔXist* haploid-diploid mixed ESCs after EpiLC

differentiation for 48 hours. *Xist* expression was detected by RNA FISH using a *Xist* probe (white). Nuclei are shown in blue (DAPI staining). The area marked by a red square in the left image is enlarged on the right. *Xist* clusters were observed in WT EpiLCs (arrowheads) but not in *ΔXist* EpiLCs.Scale bar (white), 100 μm; scale bar (red), 20 μm.

Table S1. List of oligos

Name	Sequence (5' to 3')	Application
Blimp1-F	AGC ATG ACC TGA CAT TGA CAC C	RT-PCR
Blimp1-R	CTC AAC ACT CTC ATG TAA GAG GC	RT-PCR
Dnmt3b-F	CTC GCA AGG TGT GGG CTT TTG TAAC	RT-PCR
Dnmt3b-R	CTG GGC ATC TGT CAT CTT TGC ACC	RT-PCR
Gapdh-1	AGG TCG GTG TGA ACG GAT TTG	RT-PCR
Gapdh-2	TGT AGA CCA TGT AGT TGA GGT CA	RT-PCR
Prdm14-F	ACA GCC AAG CAA TTT GCA CTA C	RT-PCR
Prdm14-R	TTA CCT GGC ATT TTC ATT GCT C	RT-PCR
Stella-F	AGG CTC GAA GGA AAT GAG TTT G	RT-PCR
Stella-R	TCC TAA TTC TTC CCG ATT TTC G	RT-PCR
Tfap2c-F	GGG CTT TTC TCT CTT GGC TGG T	RT-PCR
Tfap2c-R	TCC ACA CGT CAC CCA CAC AA	RT-PCR
Xist-F	GCT GGT TCG TCT ATC TTG TGG GTC	RT-PCR
Xist-R	TTG TTC AGA GTA GCG AGG ACT TGA AGA G	RT-PCR
Xist-P1	CCA GCC ATG TTT GCT CGT TT	Genotyping
Xist-P2	GGC GAA GGA GTA TGG CCT TT	Genotyping
Xist-gRNA1-F	CACC AAG CCA TAA GGC TTG GTG GT	gRNA
Xist-gRNA1-R	AAAC ACC ACC AAG CCT TAT GGC TT	gRNA
Xist-gRNA2-F	CACC GAC CCT TGC TGT ACT GCA AA	gRNA
Xist-gRNA2-R	AAAC TTT GCA GTA CAG CAA GGG TC	gRNA

Supplemental Experimental Procedures

Derivation of a *ΔXist* haESC line

For derivation of a *ΔXist* haESC line, a deletion of *Xist* exon 1 was engineered in a haESC line from 129S6/SvEvTac mice using the CRISPR-Cas9 system. Two guide RNAs (gRNAs) targeting *Xist* exon 1 were designed using Synthego CRISPR Design Tool (www.synthego.com). Sequences of gRNAs are listed in Table S1. The gRNAs were ligated into the pX330-U6-Chimeric_BB-CBh-hSpCas9 vector (Addgene, #42230) that was digested with BbsI restriction enzyme. Transfection of vectors and the establishment of targeted haESC lines were performed as previously described (Aizawa et al., 2020a; Aizawa et al., 2020b). Briefly, 2 Cas9/gRNA vectors, a piggyBac plasmid carrying a CAG-DsRed-IRES-hygro transgene, and a hyperactive piggyBac transposase plasmid were transfected into the haESC line using lipofectamine 2000 by following the manufacturer's protocol. Subsequently, single haploid cells expressing DsRed were isolated by cell sorting (MoFlo Astrios EQ, Beckman Coulter) after staining with 15 µg/ml Hoechst 33342 (Invitrogen) and cultured on irradiated MEFs in Serum+2i+LIF medium. After the growth of clonal single colonies, a subset of cells in each line was analyzed by flow cytometry after staining with Hoechst 33342 to select cell lines containing haploid cells and genotyped to screen cell lines for a deletion of *Xist* exon 1. Subsequently, the haploid 1n cell population of each selected haploid cell line was purified by cell sorting after Hoechst 33342 staining and was cultured on a gelatin-coated plate without MEFs in Serum+2i+LIF medium.

In vitro neural lineage differentiation

The differentiation procedure to derive neural stem cell-like cells (NSCLCs) from haESCs

was performed following a published protocol (He et al., 2017) with a few modifications as schematically summarized in Figure 4E. Briefly, at day 0 haploid-diploid mixed ESCs were plated into a Sphericalplate 5D (Kugelmeiers Ltd.) in N2B27 medium supplemented with 300 nM LDN193189 (MedChemExpress, HY-12071A), 10 µM SB431542 (Stemgent, 04-0010-10) and 20 µM Y-27632 (ROCKi; Tocris, 1254). After 3 days, the EBs were plated onto dishes coated with 50 µg/ml poly-D-lysine and 10 µg/ml laminin in NSCLC differentiation medium, which consists of N2B27 medium supplemented with 300 nM LDN193189, 10 µM SB431542, 20 µM Y-27632, 20 ng/ml EGF (Peprotech, 315-09), and 20 ng/ml FGF2 (Peprotech, 450-33). On day 5, cells were washed and NSCLC differentiation medium without Y-27632 was added. At day 7 after initial induction, the differentiated cells were dissociated, and their DNA content was measured by flow cytometry.

Karyotyping

On day 7 of germ cell differentiation, M-phase arrest of EBs was performed by culturing in PGCLC differentiation medium supplemented with 0.05 mg/ml demecolcine (Merck) for 8 hours. Subsequently, PGCLCs were sorted by FACS. Chromosome counting of PGCLCs was performed as previously described (Aizawa *et al.*, 2020a).

Genotyping

Genotyping of ESC lines was performed as previously described (Aizawa *et al.*, 2020a; Aizawa *et al.*, 2020b). Sequences of *Xist* exon 1 were obtained from PCR products through the commercial Ecoli NightSeq service (Microsynth). Primers used for genotyping are listed in Table S1.

Transcription analysis

During germ cell differentiation of haploid-diploid mixed ESCs, G0/G1/S-phase haploid and S/G2/M-phase diploid ESCs, EpiLCs, and d7 PGCLCs were sorted from ESCs, EpiLCs and d7 EBs by FACS, respectively. E12.5 gonads were harvested from 129S6/SvEvTac mouse embryos. Sex of the gonads was determined by their morphology. PGCs were identified as gonadal cells positive for both SSEA1 and integrin β3 and were sorted by FACS. RNA of each sample was extracted using the RNeasy Mini Kit (Qiagen) following the manufacturer's protocol, including an on-column DNA digest using RNase-free DNase (Qiagen). RNA concentration was determined using a NanoDrop Lite (Thermo Fisher Scientific). 100 - 500 ng total RNA was reverse transcribed using the PrimeScript RT Master Mix (Takara) according to the manufacturer's instruction. RT-PCR was performed on a 384 well format with the 480 Lightcycler instrument (Roche) using KAPA SYBR FAST qPCR KIT (Kapa Biosystems). Fold change expression was calculated using the ΔΔct method. *Gapdh* expression of G0/G1/S-phase haploid ESCs was used to normalize the transcription of G0/G1/S-phase EpiLCs and d7 PGCLCs. *Gapdh* expression of S/G2/M-phase diploid ESCs was used to normalize the transcription of S/G2/M-phase EpiLCs, d7 PGCLCs and PGCs. Primers used for transcription analysis are listed in Table S1.

Xist RNA FISH

RNA FISH was performed to analyze *Xist* expression during differentiation of haploid-diploid mixed ESCs. The *Xist* FISH probe was prepared from the ptetOP-Xist-PA plasmid with Cy3-dCTP (Amersham Biosciences) as described previously (Wutz and Jaenisch, 2000). For analysis of germ cell differentiation, G0/G1/S-phase haploid and S/G2/M-phase diploid ESCs, EpiLCs and d7 PGCLCs were sorted by FACS, respectively. For analysis of

time frame EpiLC and *Xist* deletion effect, all cells containing both haploid and diploid population were used after harvesting without cell sorting. Cells were mounted onto glass slides using a Cytospin 4 (Thermo Scientific) for 3 min at 800 or 900 rpm for haploid or diploid cells, respectively. Glass slides were immediately rinsed in PBS and washed in CSK buffer (100 mM NaCl, 300 mM sucrose, 3 mM $MgCl_2$, 10 mM PIPES pH 6.8) for 30 sec, in CSK buffer + 0.5% Triton X-100 for 2 min, and again in CSK buffer for 30 sec. Cells were fixed in 4% paraformaldehyde in PBS for 10 min and dehydrated through a series of 70%, 80%, 95%, and 100% EtOH for 2 min each. After airdrying the slides, hybridization was performed by applying probe to the cells. Probes were covered with a coverslip and sealed with rubber cement. Slides were then placed in a humidified chamber and incubated overnight at 37°C. Coverslips were removed and slides were washed in 2X SSC + formamide (50%) for 15 min at 39°C, in 2X SCC three times for 5 min each at 39°C, in 1X SSC for 10 min, and in 4X SSC with a dip (20X SSC: 3 M NaCl, 0.3 M tri-sodium citrate dihydrate in H_2O). Cellular DNA was counterstained by incubating slides in 4X SSC + 0.1% Tween + 14 mM DAPI for 1.5 min. Slides were washed with 4X SSC for 5 min. After washing, the sample was mounted in Vectashield (Vector Laboratories) and covered with a coverslip. Samples were imaged under the microscope (Axio Observer Z1, Zeiss) equipped with an ORCA-Flash4.0 camera (Hamamatsu Photonics K.K.). Images were processed using Zeiss Zen Pro 2.0 software.

X chromosome painting

X chromosome staining procedures were performed after imaging RNA FISH samples. To strip off RNA FISH dye and DAPI staining, the coverslips were removed and the glass slides were washed in 2X SSC + formamide (70%). The flask was incubated in a water bath at

85°C for 10 min. Subsequently the slides were washed three times in 2X SSC at RT for 5 min each. For detecting the mouse X chromosome, XMP X Green probe (MetaSystems Probes, D-1420-050-FI) was 1:3 diluted in Hybrisol VII (MP Biomedicals) and added to the sample. The samples were covered by coverslips, sealed with rubber cement, and put on a heat block at 75°C for 2 min. Then, the slides were placed in a humidified chamber and incubated at 37°C overnight. The next day, cover slips were removed. Glass slides were washed in 0.4X SSC at 72°C for 2 min, followed by 2X SSC, 0.05% Tween at RT for 30 sec, and then counterstained with 14 mM DAPI in 2X SSC, 0.05% Tween at RT for 90 sec. Then, slides were washed in 2X SSC at RT for 5 min. The samples were mounted in Vectashield Antifade Mounting Medium (Vector Laboratories) and covered with cover slips. The samples were imaged under the microscope (Axio Observer Z1, Zeiss) equipped with an ORCA-Flash4.0 camera (Hamamatsu Photonics K.K.). Images were processed using Zeiss Zen Pro 2.0 software.

Supplemental References

Aizawa, E., Dumeau, C.-E., Freimann, R., Di Minin, G., and Wutz, A. (2020a). Polyploidy of semi-cloned embryos generated from parthenogenetic haploid embryonic stem cells. PLoS One *15*, e0233072. 10.1371/journal.pone.0233072.

Aizawa, E., Dumeau, C.E., and Wutz, A. (2020b). Application of Mouse Parthenogenetic Haploid Embryonic Stem Cells as a Substitute of Sperm. J Vis Exp. 10.3791/61999.

He, Z.Q., Xia, B.L., Wang, Y.K., Li, J., Feng, G.H., Zhang, L.L., Li, Y.H., Wan, H.F., Li, T.D., Xu, K., et al. (2017). Generation of Mouse Haploid Somatic Cells by Small Molecules for Genome-wide Genetic Screening. Cell Rep. *20*, 2227-2237. 10.1016/j.celrep.2017.07.081.

Wutz, A., and Jaenisch, R. (2000). A shift from reversible to irreversible X inactivation is triggered during ES cell differentiation. Mol. Cell *5*, 695-705. 10.1016/s1097-2765(00)80248-8.
